# Next-generation sequencing of the athletic gut microbiota: a systematic review

**DOI:** 10.20517/mrr.2022.16

**Published:** 2023-02-23

**Authors:** Carlos Sabater, Eduardo Iglesias-Gutiérrez, Lorena Ruiz, Abelardo Margolles

**Affiliations:** ^1^Department of Microbiology and Biochemistry of Dairy Products, Instituto de Productos Lácteos de Asturias-Consejo Superior de Investigaciones Científicas (IPLA-CSIC), Paseo Río Linares s/n, Villaviciosa 33300, Spain.; ^2^Functionality and Ecology of Beneficial Microbes (MicroHealth) Group, Instituto de Investigación Sanitaria del Principado de Asturias (ISPA), Oviedo 33011, Spain.; ^3^Department of Functional Biology, Area of Physiology, Universidad de Oviedo, Avda. Julián Clavería 6, Oviedo 33006, Spain.; ^4^Traslational Interventions for Health (ITS) Group, Instituto de Investigación Sanitaria del Principado de Asturias (ISPA), Oviedo 33011, Spain.

**Keywords:** Gut microbiome, metagenomics, athletes, exercise, systematic review

## Abstract

**Aim:** There is growing evidence that physical activity modulates gut microbiota composition through complex interactions between diet and microbial species. On the other hand, next-generation sequencing techniques include shotgun metagenomics and 16S amplicon sequencing. These methodologies allow a comprehensive characterisation of microbial communities of athletes from different disciplines as well as non-professional players and sedentary adults exposed to training. This systematic review summarises recent applications of next-generation sequencing to characterise the athletic gut microbiome.

**Methods:** A systematic review of microbiome research was performed to determine the association of microbiota composition profiles with sports performance.

**Results:** Bibliographic analysis revealed the importance of a novel research trend aiming at deciphering the associations between individual microbial species and sports performance. In addition, literature review highlighted the role of butyrate-producing bacteria such as *Anaerostipes hadrus*, *Clostridium bolteae*, *Faecalibacterium prausnitzii*, *Roseburia hominis* and unidentified species belonging to *Clostridiales*, *Lachnospiraceae* and *Subdoligranulum* species in gut health and sports performance across several disciplines. Interestingly, metabolic activities of *Prevotella copri* and *Veillonella atypica* involved in branched amino acid and lactate metabolism may contribute to reducing muscular fatigue. Other microbial metabolic pathways of interest involved in carbohydrate metabolism showed increased proportions in athletes´ metagenomes.

**Conclusion:** Future research will aim at developing personalised nutrition interventions to modulate key species associated with certain components of exercise.

## INTRODUCTION

There is growing evidence that physical activity modulates gut microbiota composition^[1]^. In this sense, human microbiome is sensitive to the physiological changes associated with training, including increased intestinal permeability and altered profiles of inflammatory markers. It has been described that gut microbiota regulates the energetic balance and the inflammatory, redox, and hydration status. Specifically, physical activity may lead to an increase in microbiota diversity^[2]^. These increased microbial diversity estimators include α-diversity, indicating the number of clades in one sample within an individual^[3]^. Previous studies suggest that athlete microbiota shows higher proportions of certain members of the Firmicutes phylum and the genus *Akkermansia*^[3,4]^. The interactions between diet, exercise, and gut microbiota in athletes have been reported^[5]^. Common dietary strategies and recommendations for elite athletes are primarily based on high protein and simple carbohydrate intake and low consumption of plant polysaccharides. However, other factors that should be considered in these recommendations include the sports discipline (that may require specific physiological and metabolic demands), sex, nutrient sources with different amino acid contents, and sports periodization (involving pre- and post-training and pre- and post-competition phases). Some of these dietary recommendations may result in reduced microbiota diversity and functionality^[5,6]^. It has been reported that elite athletes suffer from psychological and gastrointestinal conditions associated with gut microbiome^[6]^. Administration of specific probiotic strains, prebiotics and synbiotic combinations could promote a healthy microbiota, which may exert a direct influence on the hypothalamus-pituitary-adrenal axis in athletes^[2,5,6]^.

On the other hand, computational tools involving machine learning have been used to predict exercise-induced alterations in the microbiota of athletes^[7]^. These pattern-recognition algorithms might be especially useful to distinguish between responders and non-responders to specific nutrients or supplements in order to develop personalised nutrition strategies aiming at improving sports performance. Similarly, machine learning could be of great interest to rationally design prebiotic interventions targeting species and strains associated with health benefits^[8]^, and perform systematic literature reviews dealing with microbial carbohydrate metabolism^[9]^. Systematic reviews in the field of gut-microbiota-brain axis study revealed the associations between physical and emotional stress during exercise in animal models. This fact may induce changes in gut microbiome including microbial clades involved in the degradation of intestinal mucus and immune response^[6]^. Other systematic reviews suggested that probiotic administration may improve athletes’ general health and performance while controlling inflammation and redox levels^[3]^. To our knowledge, no systematic review has addressed the association of gut microbes with sports performance with an emphasis on metagenomics. In this regard, shotgun sequencing shows several advantages compared to amplicon sequencing and culture depending techniques, including the taxonomic identification of non-culturable microbes at species and strain level, the recovery of microbial metagenome-assembled genomes and the study of microbial metabolic pathways [Table 1]. Therefore, this systematic review provides an overview of next-generation sequencing of the athletic microbiota and summarises the main genera and species associated with physical performance. Microbial clades associated with physical activity in non-professional players and sedentary adults are discussed.

**Table 1:** Advantages and disadvantages of culture depending techniques, amplicon and shotgun sequencing

	**Shotgun sequencing**	**Amplicon sequencing**	**Culture depending techniques**
**Advantages**	Identification of non-culturable microorganisms	Identification of non-culturable microorganisms	Possibility to perform phenotypic studies
	Taxonomic identification at species and strain level	Taxonomic identification at genus level	Low cost per sample
	Recovery of microbial metagenome-assembled genomes	Lower cost per sample	
	Study of microbial metabolic pathways	Lower concentrations of DNA needed	
**Disadvantages**	High cost per sample	Microbes cannot be identified at species and strain level	Limited to culturable microorganisms
	High concentrations of DNA needed	Microbial metabolic pathways cannot be studied	Less accurate view of microbial biodiversity

## METHODS

To select the articles included in this systematic review, an advanced search of original articles listed in two different databases (Scopus and Web of Science) was first performed. This search contained two conceptual terms involving athletes and metagenomics while excluding review articles and book articles. For this purpose, an advanced search option with boolean operators was used. The format of this initial search was adjusted for each database [Table 2]. It should be noted that word roots and synonyms were introduced in the metagenomics conceptual term in order to retrieve the maximum number of results (26 and 18 for Scopus and Web of Science, respectively).

**Table 2:** Advanced queries introduced Scopus and Web of Science databases

**Bibliographic search (*n* = 27)**	
**Database**	**Query (boolean string)**
Scopus (*n *= 26)	TITLE-ABS-KEY [(athlete AND metagen*) AND NOT (review OR chapter)]
Web of science (*n *= 18)	TI = [(athlete AND metagen*) NOT (review OR chapter)] OR AB = [(athlete AND metagen*) NOT (review OR chapter)] OR KP = [(athlete AND metagen*) NOT (review OR chapter)]

It should be noted that word roots (indicated by an asterisk) were introduced in some conceptual terms in order to retrieve the maximum number of results (26 and 18 for Scopus and Web of Science, respectively).

**Figure 1:**
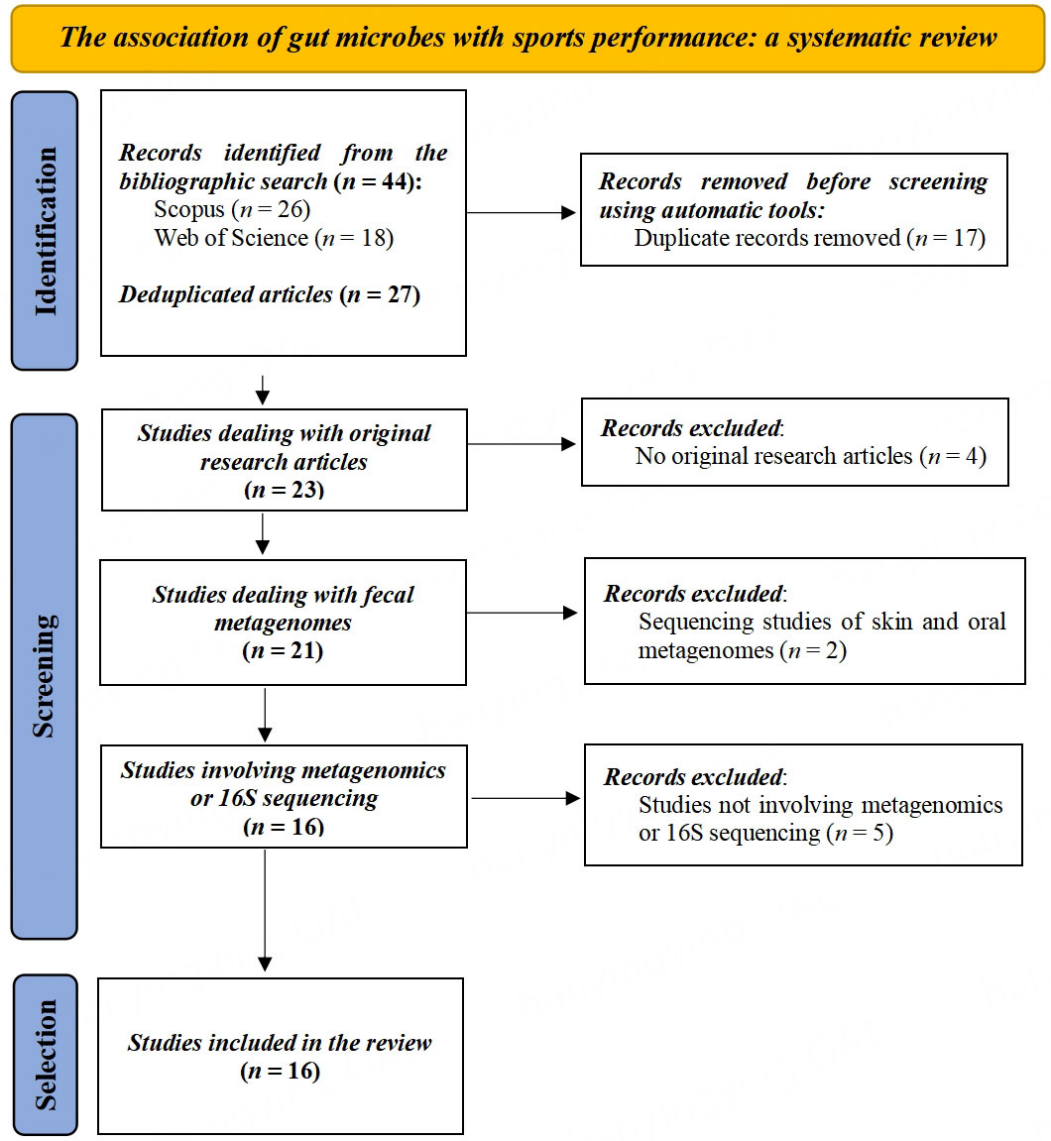
Preferred Reporting Items for Systematic reviews and Meta-Analyses (PRISMA) flow diagram illustrating the selection of articles describing the association of gut microbes with sports and exercise performance assessed by shotgun metagenomics or 16S amplicon sequencing.

Results from individual searches were merged using revtools v0.4.1 package implemented in R v3.6.2^[10]^. Duplicated articles were filtered by title using synthesisr v0.3.0 library^[11]^. Once bibliographic records were deduplicated, studies dealing with fecal samples analysed by shotgun metagenomics or 16S amplicon sequencing were selected. As a result, up to 16 original articles were retrieved. Figure 1 illustrates the Preferred Reporting Items for Systematic reviews and Meta-Analyses (PRISMA) object describing the article selection process.

Bibliographical analysis of screened articles was performed using several computational tools. These tools allow for summarising all scientific publications generated in one field to highlight the relationships between common terms and keywords. In this sense, novel research trends can be monitored by computing mathematical models like keyword co-occurrence networks^[12]^. In this regard, manuscript keywords were extracted and new keywords were generated from article titles and abstracts. These new keywords reflect those terms that are present in at least four article titles/abstracts and were generated using *Rapid Automatic Keyword Extraction *(RAKE) method implemented in litsearchr v1.0.0 package^[12]^. In addition, a co-occurrence network of article keywords and new keywords extracted from article titles and abstracts was computed using litsearchr v1.0.0 and ggrapgh v2.0.1 packages^[12,13]^.

## RESULTS

### Bibliographic analysis

A total of 16 original articles reported the applications of shotgun metagenomics or 16S amplicon sequencing for the analysis of fecal samples of athletes and individuals practicing sports [Table 3 and Supplementary Table 1]. These culture-independent techniques allow for characterising gut microbes without the need to culture them. In this regard, shotgun metagenomics offers some advantages compared to 16S sequencing, including a higher strain-level resolution, and the possibility of studying microbial genes and metabolic pathways. Selected articles were published between 2017 and 2022, highlighting the current relevance of this topic. To further investigate this emerging research trend, advanced mathematical models for text analysis were computed.

**Table 3:** Articles (*n *= 16) reporting the applications of next-generation sequencing, including both shotgun metagenomics and 16S amplicon sequencing of fecal samples on athlete microbiome research. Articles describing the association of gut microbiota with the exercise performance of non-athlete individuals were also considered*.* Some articles (*n *= 12) reported the associations between specific microbial clades and sports performance

**Database**	**Study**	**Technique**	**Participants**	**Sex**	**Age range**	***n***	**Taxa associated with sports performance**
**Sequencing experiments describing microbial taxa associated with sports performance (*n* = 12)**							
Scopus, WOS	Barton *et al.*^[14]^	Shotgun	Professional rugby players (*n *= 40) and controls (*n *= 46)	Male	-	86	*Akkermansia*, *Erysipelotrichaceae incertae sedis*
Scopus, WOS	Cronin *et al.*^[15]^	Shotgun	Healthy sedentary adults (*n *= 90) with a short-term exercise regime	Male and female	18-40	90	*Prevotella copri*
WOS	Kostic^[16]^	Shotgun	Marathon runners, ultramarathon runners, Olympic-caliber rowers and sedentary controls (*n *= 50)	Male and female	-	50	*Veillonella*
Scopus, WOS	Keohane *et al.*^[17]^	Shotgun	Athletes who completed an unsupported transatlantic row (*n *= 4)	Male	-	4	*Dorea longicatena*, *Prevotella copri*, *Roseburia hominis*, unclassified members of *Subdoligranulum*
Scopus, WOS	Scheiman *et al.*^[18]^	16S and Shotgun	Marathon athletes (*n *= 15) and sedentary controls (*n *= 10)	Male and female	-	25	*Veillonella atypica*
Scopus, WOS	Petersen *et al.*^[19]^	Shotgun	Competitive cyclists (*n *= 33)	Male and female	19-49	33	*Akkermansia*, *Bacteroides*, *Eubacterium*, *Prevotella*, *Methanobrevibacter smithii*, *Ruminococcus*
Scopus, WOS	O’Donovan *et al.*^[20]^	Shotgun	International level athletes (*n *= 37) across 16 different sports	Male and female	-	37	*Streptococcus suis*, *Clostridium bolteae*, *Lactobacillus *phage LfeInf,* Anaerostipes hadrus* in sports with moderate dynamic component. *Bifidobacterium animalis*, *Lactobacillus acidophilus*, *Prevotella intermedia*, *Faecalibacterium prausnitzii in sports with *high dynamic and low static components. *Bacteroides caccae* in sports with high dynamic and static components
Scopus	Fart *et al.*^[21]^	Shotgun	Physically active senior orienteers (*n *= 28) and community-dwelling older adults (*n *= 70)	Male and female	67-76	98	*Faecalibacterium prausnitzii*
Scopus, WOS	Kulecka *et al.*^[22]^	16S	Marathon runners (*n *= 11), cross-country skiers (*n *= 11) and healthy sedentary controls (*n *= 46)	Male and female	14-72	68	*Lachnospiraceae*, *Prevotella*
Scopus, WOS	Moitinho-Silva *et al.*^[23]^	16S	Healthy sedentary adults exposed to endurance and strength training and controls (*n *= 42)	Male and female	20-45	42	*Coprococcus*, *Parasutterella*, *Ruminococcaceae*
Scopus	Fukuchi *et al.*^[24]^	16S	Endurance athletes (*n *= 13)	Male and female	19-21	13	*Firmicutes*
Scopus	Han *et al.*^[25]^	16S	Rowing athletes (*n *= 19)	Female	12-26	19	*Firmicutes, Bacteroidetes, Proteobacteria* and *Actinobacteria* in all athletes. *Faecalibacterium* and unclassified members of *Clostridiales* and *Lachnospiraceae* in adult elite athletes. *Bacteroides* in young elite athletes
**Sequencing experiments describing athlete microbiota but no microbial taxa associated with sports performance (*n*** = **4)**							
Scopus, WOS	Genç^[26]^	Shotgun	Professional athletes (*n *= 5), amateur athletes (*n *= 5) and sedentary individuals (*n *= 5)	Male	18-24	15	-
Scopus, WOS	O’Donovan *et al.*^[27]^	16S and Shotgun	Cricket players (*n *= 21)	Male and female	-	21	-
Scopus	Axelrod *et al.*^[28]^	Shotgun	Endurance athletes (*n *= 7)	-	18-45	7	-
Scopus	Özkan *et al.*^[29]^	16S	Professional football players (*n *= 5), amateur football players (*n *= 5) and sedentary controls (*n *= 5)	Male	18-24	15	-

Full texts were available for all articles. WOS: Web of science.

Network modelling highlighted the co-occurrence of several terms in these scientific publications: dietary intake, physical activity, and taxonomic and functional profiles of the microbiota. In this regard, previous authors suggested that training modulates microbial diversity and microbial metabolism^[4,5]^. Moreover, a high degree of compliance with dietary recommendations for athletes greatly affects gut microbiota functionality and sports performance^[5,6]^. Interestingly, this mathematical model suggests the association of specific taxonomic clades with sports performance indicators such as endurance in professional athletes. It has been reported that athletes show increased microbiome diversity estimators like α-diversity as well as microbial pathways and fecal metabolite production compared to controls^[2,3,14]^.

The importance of each term represented in the co-occurrence network was calculated considering the number of other terms with which it appears. Some relevant terms highlight the role of physical activity on the health status, taxonomic and functional profiles of the gut microbiota. It should be noted that the number of microbiome studies dealing with sports performance of elite athletes based on parameters like endurance has incremented in the last five years. In contrast, studies published before tend to focus on the relationship between gut microbes and general dietary and lifestyle factors in professional athletes.

To assess the potential impact of co-variables in the network analysis, Spearman correlation coefficients between terms were calculated using caret package^[30]^. Then, terms showing correlation coefficients greater than 0.6 were determined. These terms were “intestinal microbiota”, “metabolic” and “specific”. It should be noted that these co-variables do not have a great impact on keyword relationships established by the co-occurrence network. On the other hand, synonyms and terms describing very similar concepts were excluded from the network to avoid bias.

### The association of gut microbes with sports performance

Among the original articles included in this systematic review, nine and five microbiome studies involved shotgun metagenomics and amplicon 16S sequencing, respectively. Moreover, two studies involved both metagenomics and 16S sequencing. These next-generation sequencing experiments involve microbiota analysis of fecal samples of professional athletes and physically active individuals [Table 3]. Specifically, participant recruitment involved elite athletes from several disciplines, including: rugby players^[14]^, marathon runners (professional and non-professional)^[16,18,22]^, endurance athletes^[24,28]^, competitive cyclists^[19]^, rowers^[16,17,25]^, skiers^[22]^, football and cricket players^[27,29]^, and other athletes across 16 different sports^[20]^. In addition, some studies characterised the microbiota of physically active senior orienteers^[21]^, as well as healthy but sedentary adults exposed to endurance and strength training^[23]^, and short-term exercise regime^[15]^. It should be noted that most of these studies compared microbiota profiles of athletes and physically-active participants to sedentary controls [Table 3]. Most studies included both male and female participants, while four and one focused on male and female athletes, respectively [Table 3]. It should be noted that these authors did not determine potential taxonomic differences according to the sex of individuals. In general, the age of participants comprising young and elite athletes ranged from 18 to 40.

**Figure 2:**
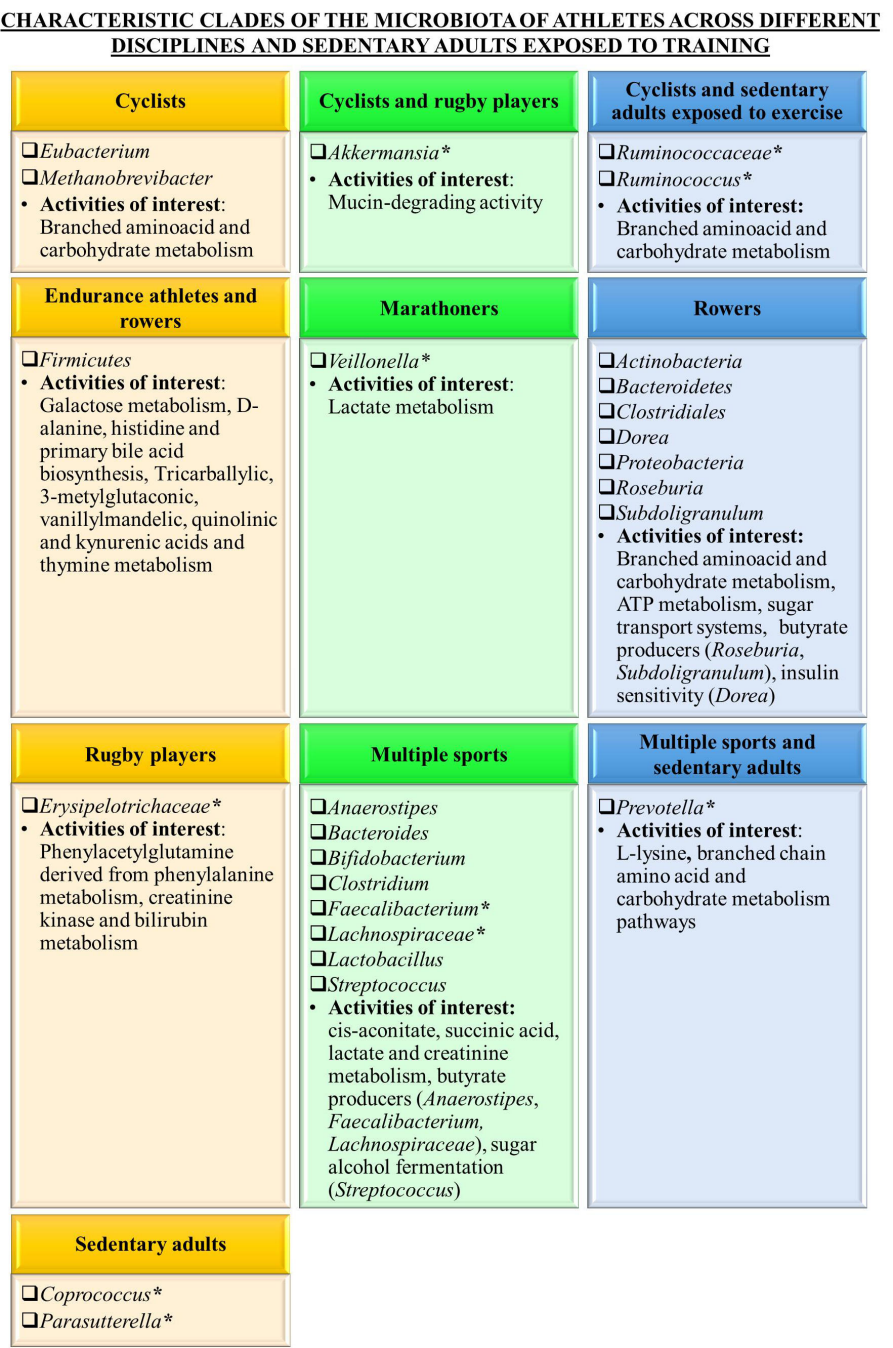
Schematic representation of characteristic microbial clades and metabolic activities with a higher representation in the microbiota of athletes across different disciplines as well as sedentary adults exposed to exercise regime. *Microbial taxa showing significantly higher abundances in the microbiota of athletes or sedentary participants exposed to exercise regime compared to sedentary controls.

A total of 12 articles described the association of specific microbial clades with sports and exercise performance [Table 3]. Figure 2 provides a schematic representation of characteristic microbial clades found in the microbiota of athletes across different disciplines as well as sedentary adults exposed to exercise regime. As can be seen, Figure 2 summarizes characteristic taxonomic profiles reported in the selected studies and presented in Table 3. In this sense, Firmicutes phylum including *Ruminococcaceae *or *Faecalibacterium *was increased in endurance athletes compared to physically inactive individuals^[1,24]^. Moreover, *Firmicutes/Bacteroidetes* ratio is correlated with obesity as well as controlling fungal occupancy in athletes^[24,31]^. In this regard, bacteria from the phyla *Firmicutes* and *Bacteroidetes* represent 90% of the gut microbiota and *Firmicutes/Bacteroidetes* ratio has been associated with maintaining normal intestinal homeostasis. Increased or decreased *Firmicutes/Bacteroidetes* ratio may lead to various pathologies. Gut microbiota unbalance usually observed with obesity, including increases in the abundance of specific *Firmicutes* or *Bacteroidetes* species^[31]^. On the other hand, high *Akkermansia* abundances were found in the microbiota of professional rugby players^[14]^ and competitive cyclists^[19]^. Low-abundance *Akkermansia* species such as *A. muciniphila* comprise mucin-degrading microorganisms that are negatively correlated with obesity and metabolic syndrome^[19]^. In contrast, *Veillonella* genus and *Veillonella atypica* might be slightly increased in marathon runners and rowers, although these increments are not statistically significant^[16,18]^. It has been suggested that lactate is the primary source of energy for *V. atypica*. Blood lactate resulting from muscle activity is associated with fatigue, and its concentration and accumulation depend on various factors, including exercise intensity, load and density. Scheiman *et al.* demonstrated in a murine model that plasma lactate may reach the intestinal lumen, where it may also be metabolized by *V. atypica*^[18]^. The relationship between physical activity levels and the prevalence of *Veillonella *involved in metabolic, protective, structural, and histological functions was also reported by Dorelli *et al.*, and Manor *et al.*^[1,32]^. The proposed mechanism of action has been proposed: *Veillonella *species metabolize lactate into acetate and propionate via the methylmalonyl-CoA pathway. Unlike *V. atypica*, many other microbes are capable of utilizing lactate through lactate dehydrogenase, but do not possess the full pathway to convert lactate into propionate. In this sense, systemic lactate resulting from muscle activity during exercise may enter the gastrointestinal lumen and is metabolized by *Veillonella *into propionate in the colon. Gut colonization of *Veillonella *promotes the Cori cycle by providing an alternative lactate-processing mechanism whereby systemic lactate is converted into SCFAs that re-enter the circulation. SCFAs are absorbed in the sigmoid and rectal region of the colon and enter circulation via the pelvic plexus, bypassing the liver and draining via the vena cava to reach the systemic circulation directly. As a consequence, microbial SCFAs derived from lactate improve athletic performance^[18]^.

Similarly, *Prevotella copri *was increased in rowers who completed an east–west transatlantic rowing race^[17]^, and sedentary adults with a short-term exercise regime involving combined aerobic and resistance training^[15]^. It has been reported that gene expression of metabolic pathways of *P. copri* involved in L-lysine is increased after ultra-endurance exercise. This essential amino acid plays a critical role in reducing muscular fatigue and contributes to muscular integrity. Furthermore, microbial derived lysine contributes to the human body protein pool^[17]^.

Other microbial clades that may be characteristic of the microbiota of athletes include unidentified taxa belonging to *Erysipelotrichaceae* in rugby players [Table 3]. These unclassified microorganisms are positively correlated to phenylacetylglutamine derived from phenylalanine. This compound is increased in athletes and associated with a lean phenotype^[14]^. However, different rugby player positions require different physiological and physical demands. High abundances of several bacterial genera (*Bacteroides*, *Eubacterium*, *Prevotella* and *Ruminococcus*) were also found in competitive cyclists compared to sedentary adults [Table 3]. These genera are correlated with branched-chain amino acid and carbohydrate metabolism pathways. It has been suggested that upregulation of branched-chain amino acids biosynthesis leads to a decrease in exercise-induced muscle fatigue and promotes muscle-protein synthesis^[19]^. Similarly, several clades like *Dorea longicatena*, *Faecalibacterium prausnitzii*, *Roseburia hominis* and unclassified species belonging to *Subdoligranulum* were increased in rowers throughout a race as well as physically active senior orienteers compared to the general older adult population^[17,21]^. *F. prausnitzii*, *R. hominis* and *Subdoligranulum* are butyrate producers that contribute to reducing gut inflammation and oxidative stress. It should be noted that butyrate is the main nutrient for colonocytes that exert an anti-inflammatory activity^[21]^. With regard to *Dorea longicatena*, this taxon is associated with insulin sensitivity^[17,33]^. In addition, high abundances of *Lachnospiraceae* were determined in marathon runners and skiers^[22]^, while *Coprococcus*, *Parasutterella* genera and *Ruminococcaceae* family were increased in physically active individuals^[23]^.

Some studies reported characteristic differences in the microbiota profiles of young and adult elite athletes in order to monitor the potential of elite athletic candidates [Table 3]. In this sense, Han *et al.* determined the prevalence of *Firmicutes, Bacteroidetes, Proteobacteria* and *Actinobacteria* in the fecal microbiota of rowing athletes^[25]^. However, unidentified species belonging to *Clostridiales* and *Lachnospiraceae*, as well as *Faecalibacterium* genus, showed higher abundances in metagenomes from elite adult participants than young elite athletes [Table 3]. These taxa are likely to produce short-chain fatty acids and may be associated with exercise-induced butyrate concentrations that may contribute to improving sports performance^[25]^. It should be noted that *Lachnospiraceae* increments are strongly associated with fiber consumptions, although general dietary recommendations for athletes are characterised by low intake of non-amylaceous polysaccharides^[5,6]^. In contrast, *Bacteroides* was enriched in the microbiota of young elite athletes [Table 3]. High abundances of oxidative stress tolerant clades may improve performance in competitive sports. The microbiota of elite athletes may also show increased proportions of microbial metabolic pathways involved in carbohydrate metabolism and multiple sugar transport systems compared to young non-elite athletes^[25]^.

Efforts to establish associations between individual microbial species and different types of physical activities have also been made [Table 3]. In this regard, *Streptococcus suis*, *Clostridium bolteae*, *Lactobacillus* phage LfeInf and *Anaerostipes hadrus *were associated with moderate dynamic components of exercise. It should be noted that dynamic components of exercise involve aerobic exercise, while static components of exercise are performed by increasing tension in a muscle while keeping its length constant. Therefore, sports disciplines can be classified into nine different categories according to the contributions (high, moderate or low) of both components^[34]^.

It has been reported that *S. suis* showed high proportions of metabolic pathways involved in the fermentation of sugar alcohols, while *C. bolteae* and *A. hadrus* may produce butyrate to promote gut homeostasis and anti-inflammatory effects^[20]^. Other clades, including *Bifidobacterium animalis*, *Lactobacillus acidophilus*, *Prevotella intermedia* and butyrate-producing species such as *Faecalibacterium prausnitzii*, were associated with high dynamic and low static components of exercise. On the contrary, *Bacteroides caccae* was enriched in athletes from disciplines involving high dynamic and static components of exercise [Table 3].

Most articles selected in this systematic review do not involve intervention studies. In this regard, characteristic patterns in the fecal microbiota of athletes were elucidated at taxonomic and functional levels [Figure 2]. In addition, some studies report differences in the metabolomic profiles of participants. In this sense, differences in the composition and functional capacity of the gut microbiome of international-level athletes from several disciplines have been determined^[20]^. It has been described that fecal microbiota samples of professional rugby players and sedentary controls show even greater separation at the functional metagenomic and metabolomic than at compositional levels^[14]^. Similarly, microbial diversity, including butyrate-producing species, increased throughout the ultra-endurance events such as transoceanic rowing races. The functional potential of bacterial species involved in specific amino and fatty acid biosynthesis also increased^[17]^. Additional studies dealing with rowing athletes report that ATP metabolism, multiple sugar transport systems and carbohydrate metabolism are enriched in the microbial community of these athletes^[25]^. Differences in functional bile acid and histidine metabolism also differentiate power athletes from sedentary controls, while galactose metabolism markers differentiate endurance athletes from controls. Moreover, D-alanine and primary bile acid biosynthesis differentiate power and endurance athletes from controls. Scheiman *et al.* and Kostic reported that every gene in a major pathway metabolizing lactate to propionate is at higher relative abundance postexercise in marathon runners^[18,16]^. On the other hand, an increment in the abundance of *Prevotella* was correlated with a number of amino acid and carbohydrate metabolism pathways in cyclists, including branched-chain amino acid metabolism^[19]^. In contrast, some studies report characteristic microbiota patterns mainly at taxonomic level, including increased abundances of *Faecalibacterium prausnitzii *in physically active senior orienteers^[21]^.

Some studies report characteristic microbial-derived metabolic profiles of athletes from different disciplines. In this regard, cis-aconitate, succinic acid and lactate in urine samples and creatinine in faeces were found to be significantly different between groups of sports. These differences were evident despite the absence of significant differences in diet^[20]^. In addition, microbial-derived SCFAs are enhanced within rugby players and their gut microbiota was predominantly correlated with creatine kinase (CK), total bilirubin and total energy intake^[14]^.

Dietary factors that may confound the association between exercise and the abundance of microbial taxa of interest were considered in several of these studies^[16,18]^. Other studies report the assessment of macronutrient intake per day to enhance inter-individual comparison^[21]^ as well as quantitative diet evaluation^[22]^. It has been reported that the versatility of the microbial community of athletes, which might affect their performance, is associated with dietary factors^[25]^. However, other studies report the lack of in-depth dietary analysis and a matching non-athlete cohort (i.e., control group comprising non-athlete participants with similar demographic characteristics and exposed to the same intervention)^[19]^.

On the contrary, three of the selected articles comprised intervention studies. In this regard, Cronin *et al.* demonstrated modest changes in gut microbial composition and function in healthy but sedentary adults exposed to short-term exercise regime, with and without concurrent daily whey protein consumption^[15]^. An association between whey protein intake and the β-diversity of the adult gut virome was found, while no major changes in the functional activity of the gut microbiota were determined, with the exception of urinary levels of trimethylamine N-oxide (TMAO) and phenylacetylglycine (PAG) excretion. Similarly, Moitinho-Silva *et al.* elucidated taxonomic differences, a significant increase in lymphocytes and a decrease in mean corpuscular haemoglobin concentration in healthy sedentary adults exposed to strength training^[23]^. No change in dietary patterns towards the end of the intervention period and after it was observed. Finally, Fukuchi *et al.* investigated the effects of both official competition and a multi-strain lactic acid bacteria-fermented soymilk extract in taxonomy and urine metabolites in endurance athletes^[24]^. Changes in urinary metabolites included a significant reduction in yeast and fungal markers, neurotransmitters, and mitochondrial metabolites including the tricarboxylic acid (TCA) cycle. Tricarballylic acid was positively correlated with the ratio of *Firmicutes*, while *Parabacteroides distasonis *was negatively correlated with several urinary metabolites, including 3-metylglutaconic, vanillylmandelic, quinolinic and kynurenic acids and thymine^[24]^.

Applications of next-generating sequencing technologies to assess the relationships between gut microbiota composition and sports performance have been summarised. Computational methods for text processing and bibliographic analysis revealed recent research trends focusing on the impact of microbial diversity and metabolic pathways on different components of physical activity. These studies highlight the role of butyrate-producing bacteria in promoting gut health and contributing to an optimal composition of the athletic microbiota.

## DISCUSSION

Mathematical modelling for text processing opens new possibilities for evidence synthesis in microbiome research. This could be of special interest to monitor research trends in emerging fields like metagenomics applied to sports science. However, this novel approach has several limitations. In this sense, the authors from selected articles may use general and unprecise terms to describe their findings, making keyword co-occurrence analysis difficult. In addition, unprecise terminology in the field of sports science, including “performance”, “exercise”, “athlete”, “elite”, is used in several articles. In this regard, authors should provide detailed participant metadata for accurate interpretation of results. Moreover, specific indicators of physical activity performance, such as endurance, should be properly described in the study design. Other limitations of current research summarised in this review include different dietary patterns among participants from different studies that show an association with gut microbiota regardless of physical activity. Similarly, different geographical origins of individuals and control groups such as non-elite athletes or sedentary adults are potential confounding variables that may be considered to assess bias in these studies.

Diet modulates the composition of the athletic gut microbiota. Long-term diets modulate gut microbiota composition. For example, protein and animal fat lead to an increase in *Bacteroides*, while simple carbohydrates are associated with *Prevotella* enrichment^[35]^. It is difficult to establish standard diet regimes due to the considerable complexity of stress responses in elite athletes. It has been proposed that gut microbiota acts like an endocrine organ by secreting serotonin, dopamine or other neurotransmitters and may control the hypothalamus-pituitary-adrenal axis in athletes resulting in the release of stress and catabolic hormones, inflammatory cytokines and microbial molecules. Therefore, dietary interventions may contribute to an improvement in sports performance^[6,36]^. However, some dietary recommendations for elite athletes are primarily based on low consumption of plant polysaccharides, which is commonly associated with reduced microbiota diversity and SCFA and neurotransmitter synthesis. In this regard, dietary recommendations for athletes should consider fiber intake to promote microbial species reduced in athlete’s gut^[6,35]^. Some studies reported a reduced diversity of the athlete’s gut microbiota that could be attributed to their higher protein intake, although there is a need for longer-term studies on athletes from different disciplines to assess the impact of diet on the athletic gut microbiota. It has been reported that protein intake was negatively correlated with diversity in marathon runners and fat intake was negatively correlated with *Bifidobacteria *in bodybuilders^[35,37]^. As explained, high-protein diets may decrease microbial diversity in endurance athletes who consume lower amounts of carbohydrates. Similarly, a decrease in SCFA-producing species has been observed in resistance athletes that follow a high-protein, low-carbohydrate, and high-fat diet. However, some dietary recommendations for endurance athletes resulting in the consumption of high amounts of both simple and complex carbohydrates in combination with training may lead to an increased abundance of *Prevotella*. This genus is associated with fiber-rich diets and participates in several amino acid and carbohydrate metabolism pathways, including branched-chain amino acid metabolism^[35]^. Interestingly, five bacterial families (*Lachnospiraceae*, *Clostridiaceae*, *Paraprevotellaceae*, *Ruminococcaceae*, and *Veillonellaceae*) were associated with physical activity in studies that controlled diet as a possible confounding factor^[1]^.

### Future trends

Future research trends may focus on the interactions of diet and gut microbial composition in the context of athletic performance as well as potential applications of pro- pre- and symbiotic supplements specifically developed for athletes. For this purpose, more studies dealing with metabolic activities and other mechanisms of action of specific clades associated with several indicators of sports performance are needed. In this regard, metagenomic sequencing enables the identification of microbial metabolic pathways showing higher abundances in the gut microbiota of athletes from several disciplines than in sedentary controls. This fact may contribute to a better understanding of the role of gut commensals in athlete physiology. In addition, further studies including a multi-omics approach (i.e. the combined analysis of metabolomic and metagenomic data) are needed to better elucidate the associations between physical activity, dietary patterns and gut microbiota. It should be noted that most articles published up to date comprise observational studies and the mechanisms of action of specific microbial clades contributing to sports performance remain unknown. In this regard, genomic sequences recovered from athlete metagenomes could be of great benefit to perform metabolic modelling in order to characterize those activities that may have a physiological impact. The integration of these techniques may lead to personalized nutrition interventions including specific nutrient recommendations aimed at improving performance by promoting certain taxa and enhancing certain metabolites of interest during exercise. Finally, more randomized controlled studies analyzing wider cohorts and controlling for potential confounding factors are needed to identify microbial species that could be used as biomarkers of sports performance.

### Conclusions

This systematic review gives a general overview of the applications of next-generation sequencing, including shotgun metagenomics and 16S amplicon sequencing, to study the athletic gut microbiota. Bibliographic analysis using computational models revealed the growing research interest in elucidating the associations between specific microbial clades and sports performance. In this sense, butyrate producers such as *A. hadrus*, *C. bolteae*, *F. prausnitzii*, *R. hominis* and unidentified species belonging to *Clostridiales*, *Lachnospiraceae* and *Subdoligranulum *may contribute to maintaining gut homeostasis in athletes. On the other hand, species involved in branched amino acid and lactate metabolism, such as *P. copri *and *V. atypica*, may reduce muscular fatigue. In addition, athletic gut microbiota shows an increase in microbial metabolic pathways involved in carbohydrate metabolism. Future research will aim at developing personalised nutrition strategies and recommendations to optimise microbiota composition profiles associated with different components of exercise (high, moderate and low static and dynamic components). Future research trends may focus on the interactions of diet and gut microbial composition in the context of athletic performance. For this purpose, multi-omic studies dealing with metabolic activities and other mechanisms of action of specific clades associated with several indicators of sports performance are needed. As a consequence, these microorganisms could be used as biomarkers of sports performance to assess the potential of athletic candidates.

## DECLARATIONS

### Availability of data and materials

PRISMA diagram is provided in Figure 1. Bibliographical analysis was performed using ggrapgh v2.0, litsearchr v1.0.0, revtools v0.4.1, synthesisr v0.3.0 packages implemented in R v3.6.2.

### Copyright

© The Author(s) 2023.
