# Supplementary material for: Next-generation sequencing of the athletic gut microbiota: a systematic review
Source: Microbiome Res Rep. 2023 Feb 23;2(1):5. doi: 10.20517/mrr.2022.16 (PMC10688803; doi:10.20517/mrr.2022.16)
Supplement: Supplementary file 1 [file mrr-2-1-5-SupplementaryMaterials.pdf]

SupplementaryMaterials

Supplementary Table 1. **Articles (*n* = 16) reporting the applications of next-generation sequencing including both shotgun metagenomics and 16S amplicon sequencing of fecal samples on athlete microbiome research. Articles describing the association of gut microbiota with *exercise performance of non-athlete individuals were also considered.* Some articles (*n* = 12) reported the associations between specific microbial clades and sports performance**

Database	Study	Technique	Participants	Sex	Age range	*n*	Taxa associated to sports performance
Sequencing experiments describing microbial taxa associated to sports performance (n = 12)							
Scopus, WOS	Barton *et al.*[14]	Shotgun	Professional rugby players (*n* = 40) and controls (*n* = 46)	Male	-	86	*Akkermansia*, *Erysipelotrichaceae incertae sedis*
Scopus, WOS	Cronin *et al.*[15]	Shotgun	Healthy sedentary adults (*n* = 90) with a short-term exercise regime	Male and female	18-40	90	*Prevotella copri*
WOS	Kostic[16]	Shotgun	Marathon runners, ultramarathon runners, Olympic-caliber rowers and sedentary controls (*n* = 50)	Male and female	-	50	*Veillonella*
Scopus, WOS	Keohane *et al.*[17]	Shotgun	Athletes who completed an unsupported transatlantic row (*n* = 4)	Male	-	4	*Dorea longicatena*, *Prevotella copri*, *Roseburia hominis*, unclassified members of *Subdoligranulum*
Scopus, WOS	Scheiman *et al.*[18]	16S and Shotgun	Marathon athletes (*n* = 15) and sedentary controls (*n* = 10)	Male and female	-	25	*Veillonella atypica*
Scopus, WOS	Petersen *et al.*[19]	Shotgun	Competitive cyclists (*n* = 33)	Male and female	19-49	33	*Akkermansia*, *Bacteroides*, *Eubacterium*, *Prevotella*, *Methanobrevibacter smithii*, *Ruminococcus*
Scopus, WOS	O'Donovan *et al.*[20]	Shotgun	International level athletes (*n* = 37) across 16 different sports	Male and female	-	37	*Streptococcus suis*, *Clostridium bolteae*, *Lactobacillus* phage LfeInf, *Anaerostipes*

							hadrus in sports with moderate dynamic component. *Bifidobacterium animalis*, *Lactobacillus acidophilus*, *Prevotella intermedia*, *Faecalibacterium prausnitzii in sports with* high dynamic and low static components. *Bacteroides caccae* in sports with high dynamic and static components
Scopus	Fart *et al.*[21]	Shotgun	Physically active senior orienteers (*n* = 28) and community-dwelling older adults (*n* = 70)	Male and female	67-76	98	*Faecalibacterium prausnitzii*
Scopus, WOS	Kulecka *et al.*[22]	16S	Marathon runners (*n* = 11), crosscountry skiers (*n* = 11) and healthy sedentary controls (*n* = 46)	Male and female	14-72	68	*Lachnospiraceae*, *Prevotella*
Scopus, WOS	Moitinho-Silva *et al.*[23]	16S	Healthy sedentary adults exposed to endurance and strength training and controls (*n* = 42)	Male and female	20-45	42	*Coprococcus*, *Parasutterella*, *Ruminococcaceae*
Scopus	Fukuchi *et al.*[24]	16S	Endurance athletes (*n* = 13)	Male and female	19-21	13	*Firmicutes*
Scopus	Han *et al.*[25]	16S	Rowing athletes (*n* = 19)	Female	Dec-26	19	*Firmicutes, Bacteroidetes, Proteobacteria* and *Actinobacteria* in all athletes. *Faecalibacterium* and unclassified members of *Clostridiales* and *Lachnospiraceae* in adult elite athlletes. *Bacteroides* in young elite athletes
Sequencing experiments describing athlete microbiota but no microbial taxa associated to sports performance (n = 4)							

Scopus, WOS	Genç[26]	Shotgun	Professional athletes (n = 5), amateur athletes (n = 5) and sedentary individuals (n = 5)	Male	18-24	15	-
Scopus, WOS	O'Donovan *et al.*[27]	16S and Shotgun	Cricket players (n = 21)	Male and female	-	21	-
Scopus	Axelrod *et al.*[28]	Shotgun	Endurance athletes (n = 7)	-	18-45	7	-
Scopus	Özkan *et al.*[29]	16S	Professional football players (n = 5), amateur football players (n = 5) and sedentary controls (n = 5)	Male	18-24	15	-

Full texts were available for all articles. WOS: Web of Science.
